# A novel in silico molecular tool for comprehensive differentiation of *Mycobacterium* species

**DOI:** 10.1038/s41598-025-89148-0

**Published:** 2025-02-10

**Authors:** Mohmoud K. Diab, Taysir Hassan A. Soliman, Amr M. Mohamed, Ibrahim E. Elsemman

**Affiliations:** 1 Department of Information Systems, Faculty of Computers and Information, Assiut University, Assiut, Egypt; 2 Department of Clinical Pathology, Faculty of Veterinary Medicine, Assiut University, Assiut, Egypt; 3 Department of Molecular Biology, Molecular Biology Research and Studies Institute, Assiut University, Assiut, Egypt

**Keywords:** Infectious-disease diagnostics, Bioinformatics

## Abstract

The Identification of various mycobacterial species is critical for understanding their pathogenicity and epidemiology. Despite the existence of several established methods for identifying mycobacterial species, each of these methods has several significant limitations, including high costs, substantial time demands, and a restricted ability to detect a wide range of recoverable species. This study presents an in silico method using restriction fragment length polymorphism (RFLP) to differentially identify 75 clinically important mycobacterial species.The present investigation employed specific primer combinations to identify and generate a distinct hypervariable sequence across the ribosomal RNA gene. This unique sequence using appropriate restriction enzyme digestion followed by gel electrophoresis enabled the creation of highly precise and distinct patterns or profiles for each of the 75 medically relevant *Mycobacterium* species, including members of closely related *Mycobacterium* complex groups. This approach can quickly and reliably identify mycobacterial species, allowing for more timely treatment decisions and contributing to beneficial epidemiological investigations.

**Supplementary Information:**

The online version contains supplementary material available at 10.1038/s41598-025-89148-0.

## Introduction

Mycobacterial infections pose significant challenges in public health management. The genus *Mycobacterium* encompasses a remarkably diverse range of bacterial species, comprising over 200 members, of which more than 60 exhibit clinical significance^1,2^. We can broadly categorize *Mycobacterium* into two main groups: *Mycobacterium tuberculosis* complex (MTBC) and nontuberculous mycobacteria (NTM)^2^. MTBC includes several closely related species, the most prominent of which is *Mycobacterium tuberculosis*, the causative agent of tuberculosis (TB), which is a leading infectious disease responsible for an estimated 1.3 million deaths annually^3^. The NTM group comprises more than 150 distinct species, many of which are medically significant and can cause a range of infections, such as pulmonary disease, skin infections, and disseminated infections^4–6^. In general, mycobacterial infections pose significant challenges in clinical practice because of their inherent drug resistance and complex treatment regimens. The typing of mycobacterial species is crucial for accurate diagnosis, understanding disease patterns, and implementing targeted control measures that enable effective public health interventions^3^. However, species identification can be challenging because of genetic diversity and overlapping characteristics^7,8^. Previous assays involving DNA amplification and subsequent sequencing, restriction analysis, and probe identification of specific genomic targets have been described as effective tools for the identification of *Mycobacterium* species. These assays use genetic elements such as 16S rDNA, ITS, and hsp65, which can provide potential information for species differential identification^9,10^. The MicroSeq 500 16S rDNA system^11^, the INNO-LiPA MYCOBACTERIA v2 system^12^, the MycoAlign system^13^, the PRA-hsp65 system^14^, and the combined rpoB duplex PCR and hsp65 PCR-RFLP^15^ were some of these tools. They worked on different genetic elements and helped identify different types of *Mycobacterium*. However, each assay had its own limitations. These limitations range from limited coverage to expensive sample costs, long turnaround times, lack of automation, and the need for specialized experts. In addition, none of the tests described so far could identify differences between members of the MTBC group because the targeted sequences were extremely similar in all members of this group.

Whole-genome sequencing (WGS) has emerged as a powerful molecular tool for examining mycobacterial genomes. Bioinformatics tools play a crucial role in the analysis and manipulation of large genetic sequences, particularly in studies involving *Mycobacterium* species WGS data^16^. PCR-RFLP, or Polymerase Chain Reaction-Restriction Fragment Length Polymorphism, is an easy and inexpensive molecular method that can amplify a specific target sequence and use a specific restriction enzyme to identify mutations and genotypes. The bioinformatics tools WebCutter, NEBCutter^17^, and REHUNT^18^ can predict the size and number of fragments after applying a specific restriction enzyme to a specific sequence. Additionally, the RFLP was used to extract features from the simulation of restriction enzyme digestion, and machine learning methods can further use these features to classify pathogen genome sequences^19^. However, these classifiers require whole-genome sequences, which are difficult and nonpracticalin clinicalsettings^20^, and they also have some limitations in sequence technologies^21^. The authors believe that a new tool should be developed to simulate the whole PCR-RFLP process in a computer. This tool should be able to identify specific target sequences from whole-genome sequences and specific restriction enzymes to identify differences between species in an important clinical bacterial family, like *Mycobacterium*.

The current investigation hypothesized that mycobacterial genomes contain unique conserved targets with diverse sequences that can provide precise identification and differentiation between their types using suitable molecular assays. The present study proposes an in silico approach that identifies a unique conserved target within the rRNA gene of the mycobacterial genome, enabling the differential identification of different clinically important *Mycobacterium* species. In the proposed assay, an RFLP analysis of the pan-mycobacterial sequence using a special restriction enzyme is required to create gel electrophoresis patterns specific to each species. A successful implementation of this PCR-based method would provide a rapid and reliable method for identifying mycobacterial species, enabling prompt and appropriate treatment decisions.

## Methods

### Data preparation

We downloaded 75 complete mycobacterial genomes with annotated files (GTF files) from the National Center for Biotechnology Information (NCBI) database (Table 1). This study searched for a specific target that satisfies three conditions: (1) the target located between two conserved regions (at least 20 bp), (2) the target has a high variable sequence, and (3) the restriction enzyme recognition sequences within the selected target can provide for differential identification between the 75 recruited *Mycobacterium* species. We constructed a set of 20-mers from different *Mycobacterium* species, including *M. tuberculosis*,* M. leprae*,* M. avium*,* M. gordonae*,* M. abscessus*,* M. marinum*,* and M. chelonae*, to identify conserved regions in the 75 mycobacterial genomes. For each genome, we selected the first 20 nucleotides and applied a sliding window of *n* nucleotides from the first position to identify the *n* 20-mers. Because the average length of the genome sequence is approximately 5 million base pairs, this process generates a list of approximately 35 million 20-mers for these seven genomes. The selection was then narrowed to involve only the 20-mers that appeared in all seven genomes. The selected list was re-filtered against the 75 investigated mycobacterial genomes to obtain the core 20-mers set of the investigated mycobacterial genomes. We mapped the final list of 20-mers common to the 75 genomes back to the genome sequence to identify the target containing these 20-mers.

**Fig. 1:**
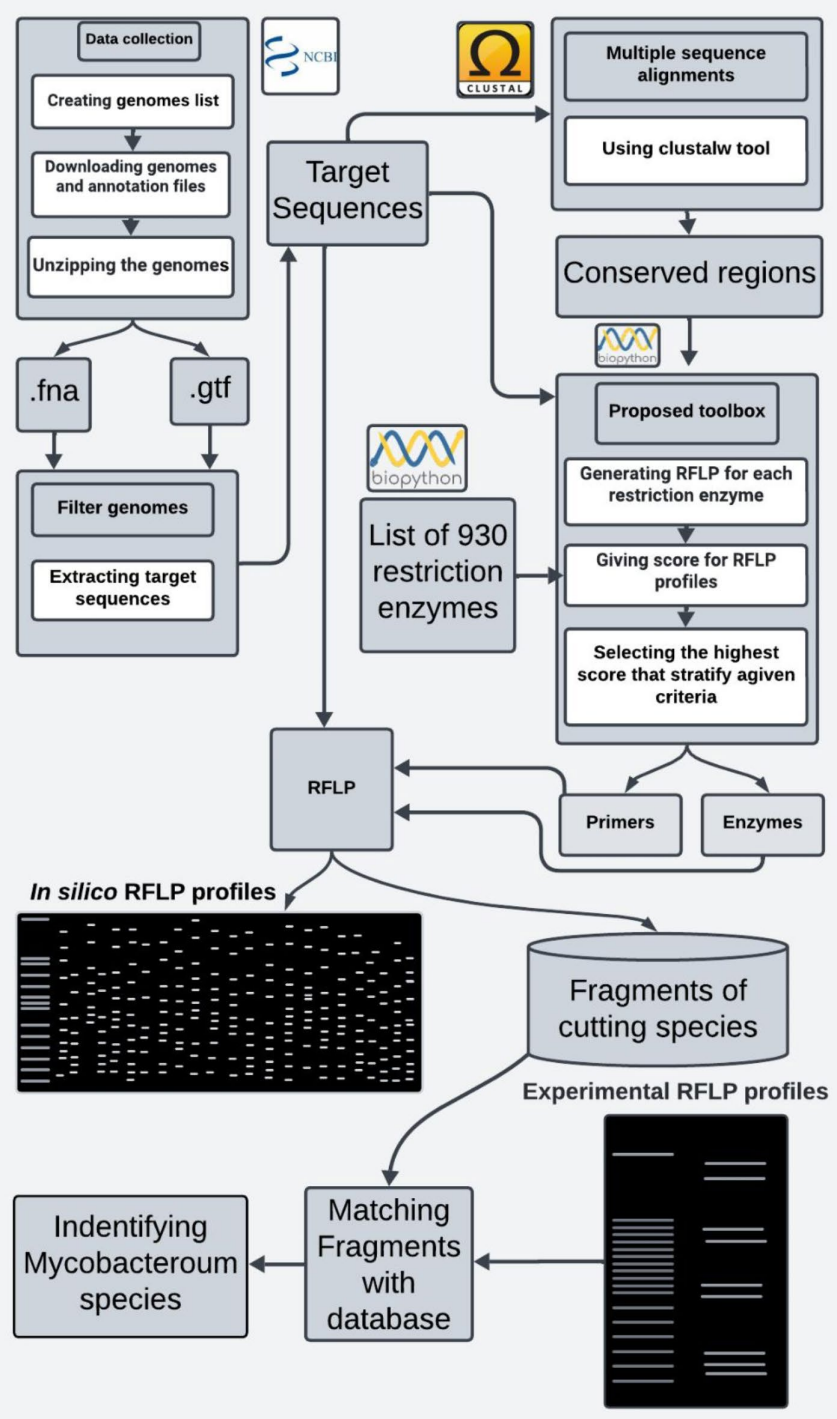
Pipeline proposed to simulate the in silico process of PCR-RFLP to discriminate among the maximum number of mycobacterial species.

We used the Clustal Omega Toolbox^22^ for multiple sequence alignment and target set analysis. The default parameters were used to identify conserved and diverse sequences among the various recruited sequences. This allowed us to identify potential pan-mycobacterial primer sets and maps of restriction enzymes, which we could use in the PCR-RFLP technique to distinguish between different mycobacterial species (Fig. 1).

**Table 1:** Accession numbers of the downloaded mycobacterial genomes.

ID	Species	ID	Species
NZ_CP034181. 1	*M. abscessus*	NZ_CP062008. 1	*M. mucogenicum*
NC_015758. 1	*M. africanum*	NZ_AP022583. 1	*M. noviomagense*
NZ_CP018019. 1	*M. avium*	NZ_AP022562. 1	*M. novum*
NZ_LR130759. 1	*M. basiliense*	NZ_LDPP01000006	*M. nebraskense*
NZ_AP022606. 1	*M. branderi*	NZ_CP074376. 1	*M. neoaurum*
NZ_CP039850. 1	*M. bovis*	NZ_CP011530. 1	*M. immunogenum*
NZ_CP016401.1	*M. caprae*	NZ_CP085945. 1	*M. intracellulare*
NC_015848.1	*M.canettii*	NZ_CP089224. 1	*M. ostraviense*
NZ_AP022613. 1	*M. conspicuum*	NZ_CP092427. 2	*M. rufum*
NZ_AP022569. 1	*M. cookii*	NZ_LT629971. 1	*M. rutilum*
NZ_CP007220. 1	*M. chelonae*	NZ_CP025546. 1	M. paragordonae
NZ_CP020809. 1	*M. dioxanotrophicus*	NZ_AP024251. 1	*M. paraintracellulare*
NZ_AP022576. 1	*M. florentinum*	NZ_CP033688. 1	*M. paratuberculosis*
NZ_CP081673. 1	M.farcinogenes	NZ_CP080333. 1	*M. pallens*
NZ_CP011269. 1	*M. fortuitum*	NZ_AP022619. 1	*M. paraseoulense*
NZ_CP038799. 1	*M. frederiksbergense*	NZ_CP092488. 2	*M. paraterrae*
NC_014814. 1	*M. gilvum*	NZ_AP022614. 1	*M. parmense*
NZ_CP092364. 2	*M. goodii*	NZ_AP024828. 1	*M. senriense*
NZ_CP070973. 1	*M. gordonae*	NZ_AP022573. 1	*M. saskatchewanense*
NZ_CP011883. 2	*M. haemophilum*	NZ_CP081000. 1	*M. senegalense*
AP024310. 1	*M. heckeshornense*	NZ_CP070349. 1	*M. septicum*
NZ_AP022615. 1	*M. heidelbergense*	NZ_AP018164. 1	*M. shigaense*
NZ_LR026975. 1	*M. hassiacum*	NZ_CP054795. 1	*M. smegmatis*
CP080997. 1	*M. heraklionensis*	NZ_CP046600. 1	*M. spongiae*
NZ_CP080998. 1	*M. holsaticum*	NZ_AP022582. 1	*M. seoulense*
NC_022663. 1	*M. kansasii*	NZ_AP022575. 1	*M. shinjukuense*
NZ_CP045081. 1	*M. kubicae*	AP022572	*M. shottsii*
NZ_AP022581. 1	*M. lacus*	NZ_AP022568. 1	*M. simiae*
NZ_AP022586. 1	*M. litorale*	NZ_AP018165. 1	*M. stephanolepidis*
NZ_CP029543. 1	*M. leprae*	NZ_AP022587. 1	*M. stomatepiae*
NZ_CP083405. 1	*M. lepromatosis*	NC_015576.1	*M. sinensis*
NZ_CP080999. 1	*M. malmoense*	NC_000962. 3	*M. tuberculosis*
AP022590. 1	*M. mantenii*	NZ_LT906483. 1	*M. thermoresistibile*
NZ_CP058277. 1	*M. marinum*	NZ_CP085200. 1	*M. ulcerans*
NZ_AP022567. 1	*M. mageritense*	NZ_CP011491. 1	*M. vaccae*
NZ_LR882499. 1	*M. microti*	NC_008726. 1	*M. vanbaalenii*
NZ_AP022617. 1	*m. monacense*	NZ_CP059165. 1	*M. vicinigordonae*
CP023147. 1	*M. marseillense*		

### Generation of in silico RFLP profiles

The primary objective of the selected targets was to create a distinct RFLP profile or pattern for each of the 75 mycobacterial species studied, achieved through the use of diverse primer sets and restriction enzymes. For this purpose,. we used BioPython^23^, specifically the library Restriction (from Bio.Restriction import *). The library Restriction includes 930 restriction enzymes, each coded as a class. The enzyme class contains the function Catalyze, which takes a sequence and applies the cutting rule to the input sequence (fragment = Enzyme. Catalyze(sequence)). The output of the function Catalyze is a list of fragments cut from this restriction enzyme. Additionally, we selected candidate primers based on their 22–30 nucleotides and their placement in two conserved regions, which were identified through multiple sequence alignment analyses. Finally, we applied the list of 930 restriction enzymes in the library to obtain targets from each identified primer set. The generated RFLP profiles were then subjected to scoring criteria for evaluation.

### Scoring criteria for selected primers and enzymes

We applied three different constraints: scoring, uniqueness in mycobacterial genomes, and matching *Tm* values, to determine the ideal primer set and restriction enzyme combination that could produce distinct PCR-RFLP profiles for all 75 investigated mycobacterial genomes.

### Score constraint

The score constraint selects the maximum score that can discriminate the 75 species based on the restriction enzyme. We developed a scoring function to calculate the percentage of differences in each RFLP profile. When the difference between the lengths of the two bands exceeded 50 nucleotides (nt), we classified them as different, from the next equation:

$$\:\left|{a}_{i}-{b}_{i}\right|=\left\{\begin{array}{cc}0,&\:\left|{a}_{i}-{b}_{i}\right|<50\\\:1,&\:\left|{a}_{i}-{b}_{i}\right|\ge\:50\end{array}\right.\:\:\:\:\:\:\:$$

We set a threshold of 50 nt to match the lowest observable base pair value in the used ladder. We used the previous equation to compute the distinction between two species *A* and *B* with fragments *a* and *b*, respectively, we assume zero for empty band fragments, and *N* is the maximum length of *a* and *b*, as follows:

1 $$\:DIF\left(A,\:B\right)={\sum\:}_{i=1}^{N}\left|{a}_{i}-{b}_{i}\right|\:\:$$

This led to the calculation of differences among the 75 mycobacterial species for different primer sets and restriction enzyme combinations as follows:

2 $$\:\:Score({FP}_{i},{RP}_{j},{E}_{K}))=\sum_{x=1}^{M}\sum_{z=x+1}^{M}DIF\left({A}_{x},\:{B}_{z}\right)$$

where *FP*_*i*_ ranges from 1 to the number of forward primers, *RP*_*j*_ ranges from 1 to the number of reverse primers, $\:{E}_{K}$ ranges from 1 to the number of enzymes, *M* is the number of the investigated species in this study. $\:DIF\left({A}_{x},\:{B}_{z}\right)$ measures the difference between two species: $\:{A}_{x}$ and $\:{A}_{z}$ from Eq. (1). To determine the highest score in Eq. (2), we iterated over all possible combinations of forward primers, reverse primers, and enzymes and selected the maximum score as follows:

3 $$\:bes{t}_{score}=max\left(Score\right({FP}_{i},{RP}_{j},{E}_{k}\left)\right)$$

### Evaluation of mycobacterial specificity of selected primer sets

To estimate the specificity of the selected primer set, we downloaded 5,042 complete bacterial reference genomes from the NCBI assembly database. This set of genomes contains 1,568 distinct genera from different families. This method was performed to ensure the specificity of the selected primer set to the *Mycobacterium* genus and not to other non-mycobacterial genera. Therefore, we downloaded complete bacterial reference genomes from all available genera. In the presence of any cross-match with non-mycobacterial genera, their restriction pattern profiles were evaluated using selected restriction enzymes to validate their distinction from the mycobacterial restringing profiles in our database.

### Melting-temperature constraint

Calculating the melting temperature (Tm) of a primer set provides several advantages for molecular biology and PCR experiments. It helps to design efficient and specific primers, to optimize PCR conditions, and to evaluate primer quality. We used the NEB Tm Calculator website ( https://tmcalculator.neb.com/) to calculate Tm for a primer set by entering the nucleotide sequences of suggested forward and reverse primers in the input fields labeled “Forward Primer” and “Reverse Primer.” The salt concentration was set to 50 mM (the default value). Based on the provided sequences, the NEB Tm Calculator displays the melting temperature (Tm) of both forward and reverse primers in each primer set.

### Species identification score

We designed a tool to read PCR-RFLP-generated profile pattern images to identify species using our custom database. The tool is designed to allow users to manually detect bands. We designed a second score function to compute the similarity between an unknown species (A), which is represented as the user’s selected bands, and any species in our custom database (B). Both A and B have fragment lists a and b, respectively, where the lengths of a and b are n and m, respectively. First, we computed the match and mismatch scores between the theoretical and experimental bands, $\:{a}_{i}$, $\:{b}_{i}$, respectively, as follows:

4 $$\:match\left(\left|{a}_{i}-{b}_{i}\right|\right)=\left\{\begin{array}{cc}1,&\:\left|{a}_{i}-{b}_{i}\right|\le\:20\\\:0,&\:\left|{a}_{i}-{b}_{i}\right|>20\end{array}\right.\:\:$$

5 $$\:mismatch\left(\left|{a}_{i}-{b}_{i}\right|\right)=\left\{\begin{array}{cc}0,&\:\left|{a}_{i}-{b}_{i}\right|\le\:20\\\:1,&\:\left|{a}_{i}-{b}_{i}\right|>20\end{array}\right.\:$$

We then computed the match and mismatch scores between species *A* and *B* by counting the number of matched and mismatched bands between the species’ bands as follows:

6 $$\:Match\:\left(A,B\right)=\sum_{i=1}^{N}match\:\left(\left|{a}_{i}-{b}_{i}\right|\right)$$

7 $$\:Mismatch\:\left(A,B\right)=\sum_{i=1}^{N}mismatch\:\left(\left|{a}_{i}-{b}_{i}\right|\right)$$

Finally, we computed the similarity score *SIM (A*,* B)* between the species A and B, as follows:

8 $$\:SIM\left(A,B\right)=\:\frac{Match\:\left(A,B\right)-Mismatch\:\left(A,B\right)\:}{Max(n,m)}\:$$

After comparing the unknown entry profile against custom species-specific profiles, the highest score similarity would be selected as species identification of the unknown entry.

### Validation

Initially, to validate the specificity of the selected primer-sets for *Mycobacterium* species, selected primer-sets were examined for their ability to generate selected targets from a wide set of 5,042 complete bacterial reference genomes, which encountered 1,564 different bacterial genera, including *Mycobacterium*. The Clustal Omega Toolbox^22^ and ITOL toolbox^24^ were used to draw the phylogenetic tree of the generated targets from the examined genomes to study their relatedness.

As the proposed assay was generated based on sequence data obtained from 75 NCBI reference mycobacterial genomes, two levels of in silico validation were used to test the integrity of the proposed assay. The first validation was performed against a larger list (942 whole genomes) from different reference mycobacterial isolates assembled by NCBI. We discarded whole genomes with no species associations (*Mycobacterium* Spp). We finally used Eq. (8) to identify the closest species by estimating the similarity score.

The second level of validation was performed against raw sequencing files for mycobacterial isolates from the ENA database (Table 2). These included 203 isolates of *M. tuberculosis*, 21 isolates of *M. bovis*, 43 isolates of *M. leprae*, 8 isolates of *M. ulcerans*, 6 isolates of *M. kansasii*, *M. chelonae*, *M. simiae*, *M. mantenii*, and *M. malmoense*, and 4 isolates of *M. simiae* and *M. neoaurum*. The FASTQ files were downloaded and preprocessed via trimming. We then performed genome assembly using SPAdes-3.15.2. We extracted the target sequence from the *scaffold.fasta* file that SPAdes generated^25^. We followed previously described instructions^26^. Finally, we applied in silico digestion to the extracted target and used Eq. (8) to identify the species.

**Table 2:** Accession numbers from the ENA database for the FASTQ file of *Mycobacterium* species used to validate the proposed primer set and restriction enzyme.

Species	ENA Accession number	Number of FastQ Files
*M. tuberculosis*	PRJNA1028254	44
	PRJEB69682	159
*M. bovis*	PRJNA494982	21
*M. leprae*	PRJNA317287	43
*M. Ulcerans*	PRJNA743744	4
*M. kansasii*	PRJNA30907	8
*M. chelonae*	PRJEB9485	6
*M. simiae*	PRJDB7717	6
*M. mantenii*	PRJDB7717	6
*M. malmoense*	PRJNA747101	6
*M. neoaurum*	PRJNA598292	4

## Results

### Location of specific target

The list of 75 genomes revealed that the rRNA gene contained 1870 conserved 20-mers. There were no other conserved 20-mers found anywhere else in the mycobacterial genomes (Table S1). This result indicates that the rRNA gene is important for designing conserved primers for *Mycobacterium* species.

### Selection of candidate primers that fulfill the target criteria

Multiple alignment sequence analysis using the Clustal Omega toolbox identified 150 conserved primer sets, with each set having a distance greater than 3000 nucleotides from the selected rRNA gene target. In Silico RFLP profiles of targets generated by each set of identified primers were generated using a panel of 930 restriction enzymes. The constraint allowed the selection of profiles with a distance between any two consecutive fragments greater than or equal to 50 nt, as in Eq. (1). This step resulted in the selection of a list of 58 primer sets with 8 different restriction enzymes (Table S2). Further sequence analysis was performed to select a combination of primer sets and restriction enzymes to generate a unique species-specific profile for each of the 75 investigated mycobacterial genomes. As a result, we identified one primer set with two restriction enzymes that can generate a distinct profile for discrimination among the 75 investigated *Mycobacterium* species.

### The proposedIn silico PCR-RFLP assay for identification of *Mycobacterium* species

**Fig. 2:**
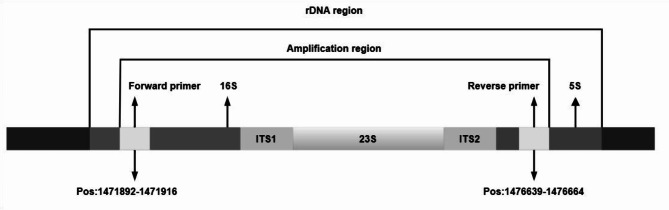
Description of the PCR target used to discriminate 75 *Mycobacterium* species. The positions are for *M. tuberculosis*.

**Fig. 3:**
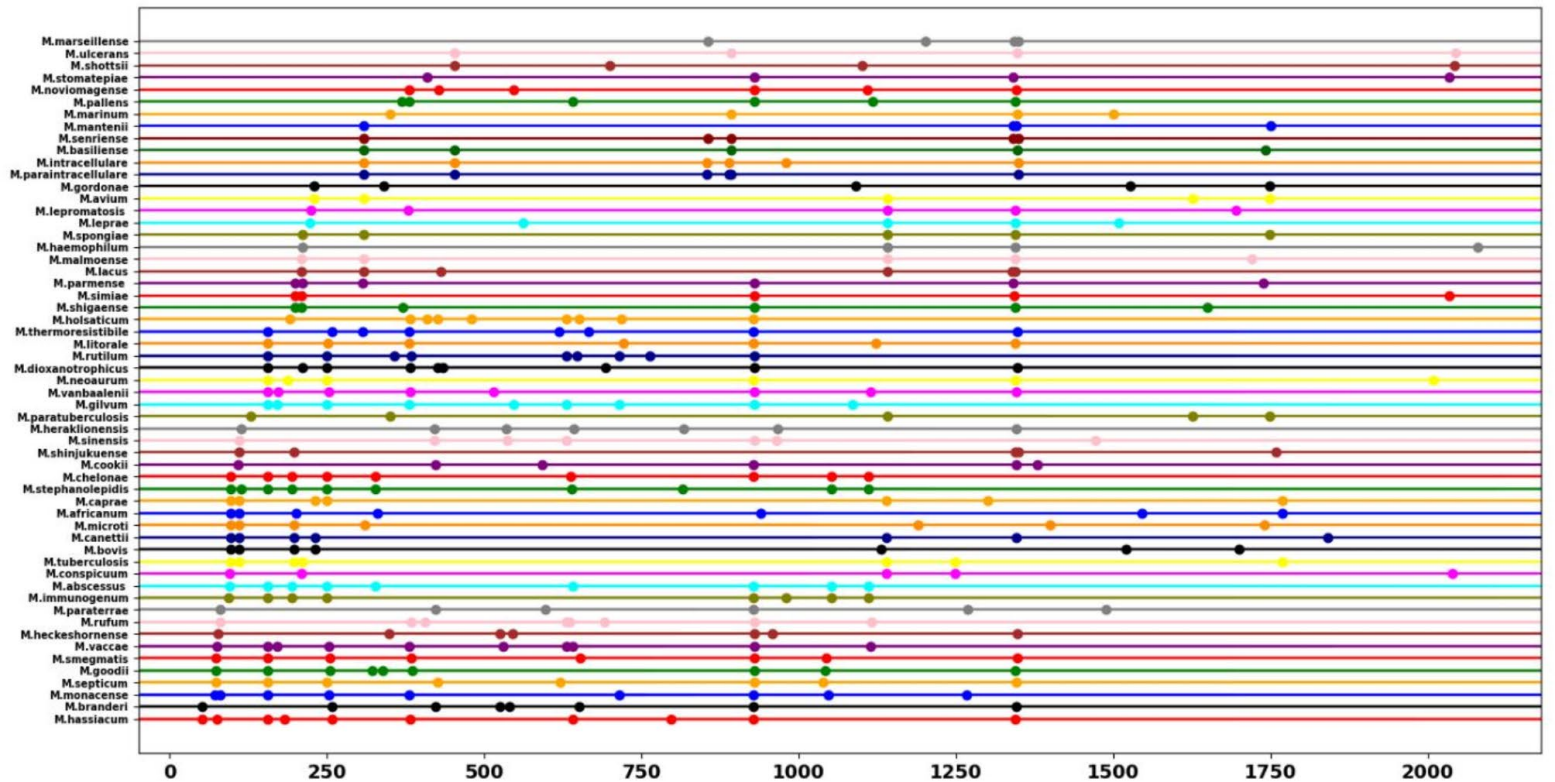
In silico profile of the TSP45I restriction enzyme discriminates among 57 *Mycobacterium* species. The point represents the fragments resulting from digestion.

**Fig. 4:**
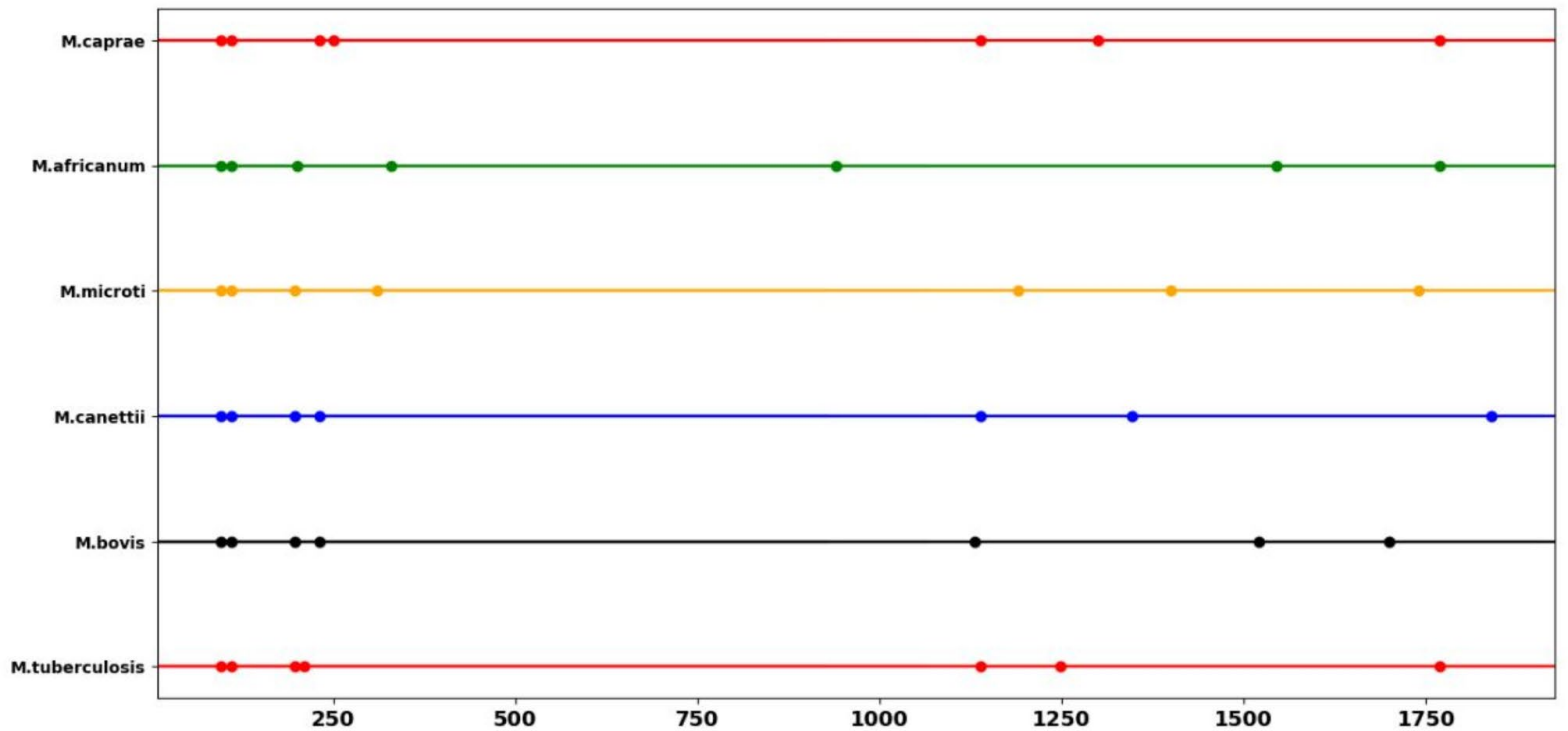
In silico profile of the TSP45I restriction enzyme discriminates among MTBC members.

**Fig. 5:**
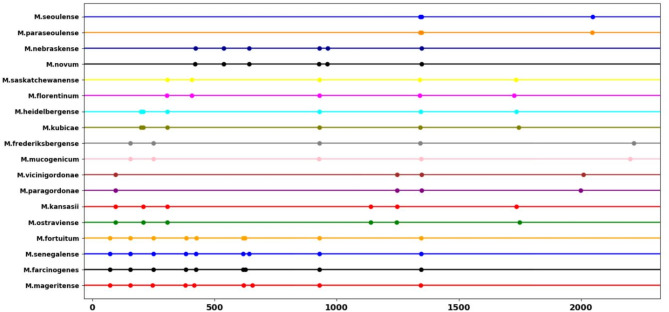
In silico profile of the TSP45I restriction enzyme in 18 *Mycobacterium* species with similar restriction patterns.

**Fig. 6:**
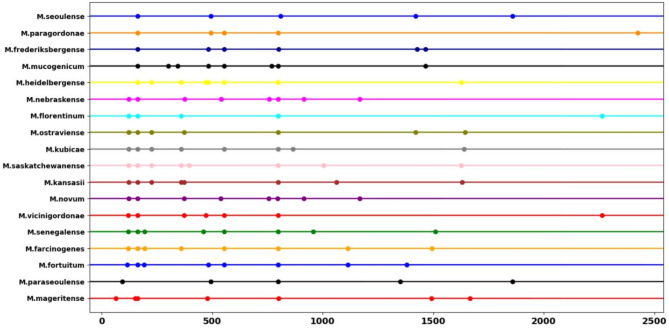
In silico profile of the BsaWI restriction enzyme discriminates among 18 *Mycobacterium* species. The point represents the fragments resulting from the digestion process.

The proposed assay included one primer set and two restriction enzymes that fulfilled the required constraints, allowing for complete discrimination between the 75 clinically important *Mycobacterium* species investigated (Table 3). The primer set comprised the forward primer, 5’-TGCTTAACACATGCAAGTCGAACG-3’, which aligns with the sequence within the 16s region, and the reverse primer, 5’-GTTTCCCGCTTAGATGCTTTCAG − 3’, which aligns with the sequence within the 5S region of the rRNA gene (Fig. 2, Table S5). Additionally, we selected two restriction enzymes to complement the previously described primer set, generating specific patterns that enabled accurate differential identification of the investigated mycobacterial species (Table 3). Tsp45I discriminated only between the 57 genomes of the 75 *Mycobacterium* species (Fig. 3). Interestingly, these included discrimination between members of the MTBC (Fig. 4). The remaining 18 species did not exhibit distinct patterns (Fig. 5). Therefore, a second restriction enzyme (BsaWI) was selected for its ability to discriminate between the remaining 18 *Mycobacterium* species (Fig. 6). The TSP45I enzyme belongs to the Type IIS restriction enzyme family. TSP45I specifically recognizes the sequence 5’-GTSAC-3’, where S is (C, G), and cleaves it to generate two DNA fragments. BsaWI is a restriction endonuclease that belongs to the Type II restriction enzyme family. It specifically recognizes the sequences 5’-ACCGGA-3’, 5’-ACCGGT-3’, 5’-TCCGGA-3’, 5’-TCCGGT-3’ and cleaves them between the two cytosine residues.

**Table 3:** Fragments size of in silico PCR-RFLP profiles of the 75 mycobacterial species.

Species	RFLP fragment size	Enzyme
*M. abscessus*	95 / 156/ 194 / 250 / 327/ 640 / 928 / 1051 / 1111	TSP45I
*M. africanum*	96 / 110 / 200 / 330 / 940 / 1545 / 1768	TSP45I
*M. avium*	229 / 308 / 1140 / 1626 / 1748	TSP45I
*M. basiliense*	308 / 452 / 892 / 1347 / 1742	TSP45I
*M. branderi*	52 / 257/ 423 / 525 / 539/ 651 / 928 / 1346	TSP45I
*M. bovis*	96 / 110/ 198 / 231 / 1139 / 1520 / 1700	TSP45I
*M. caprae*	96 / 110 / 250 / 231 / 1130 / 1300/ 1768	TSP45I
*M.canettii*	96 / 110 / 198 / 231 / 1139 /1346 / 1840	TSP45I
*M. conspicuum*	95 / 209/ 1139 / 1248 / 2039	TSP45I
*M. cookii*	109 / 423 / 592 / 928 / 1346 / 1379	TSP45I
*M. chelonae*	96 / 156/ 194 / 250 / 327 / 637 / 928 / 1051 / 1111	TSP45I
*M. dioxanotrophicus*	156 / 210 / 250 / 382 / 426 / 434 / 692 / 929/ 1347	TSP45I
*M. florentinum*	123 / 164 / 361 / 798 / 3263	BsaWI
*M. farcinogenes*	121 / 164 / 195 / 361 / 554 / 799 / 1115 / 1495	BsaWI
*M. fortuitum*	116 / 164 / 193 / 482 / 554/ 799 / 1115 / 1381	BsaWI
*M. frederiksbergense*	164 / 482 / 554 / 801 / 1428 / 1464	BsaWI
*M. gilvum*	156 / 170 / 250 / 381 / 546 / 630 / 715 / 929 / 1085	TSP45I
*M. goodii*	73 / 156 / 254 / 321 / 339 / 385 / 929 / 1042 / 1343	TSP45I
*M. gordonae*	229 / 340 / 1090 / 1526 / 1748	TSP45I
*M. haemophilum*	210 / 1140 / 1344 / 2079	TSP45I
*M. heckeshornense*	77 / 349 / 525 / 545 / 929 / 957 / 1348	TSP45I
*M. heidelbergense*	164 / 225 / 360 / 471 / 484 / 554/ 799/ 1629	BsaWI
*M. hassiacum*	52 / 75 / 156 / 182 / 258 / 382 / 640 / 796/ 928 / 1343	TSP45I
*M. heraklionensis*	113 / 421 / 534 / 642 / 817 / 966 / 1345	TSP45I
*M. holsaticum*	190/ 382/ 408/ 426/ 479/ 630/ 650/ 718/ 928	TSP45I
*M. kansasii*	123 / 164 / 225 / 360 / 373 / 798 / 1063 / 1631	BsaWI
*M. kubicae*	123 / 164 / 225 / 361/ 554/ 799 / 866 / 1639	BsaWI
*M. lacus*	209 / 308 / 430 / 1140 / 1338 / 1344	TSP45I
*M. litorale*	156 / 251 / 381 / 721 / 928 / 1122 / 1344	TSP45I
*M. leprae*	223 / 562 / 1140 / 1344 / 1508	TSP45I
*M. lepromatosis*	225 / 378 / 1141 / 1344 / 1694	TSP45I
*M. malmoense*	209 / 308 / 1140 / 1344 / 1720	TSP45I
*M. marinum*	350 / 892 / 1348 / 1500	TSP45I
*M. mantenii*	308 / 1341 / 1346 / 1750	TSP45I
*M. mageritense*	65 / 153 / 164 / 479 / 801 / 1492 / 1667	BsaWI
*M. microti*	96 / 110 / 198 / 310 / 1190 / 1400 / 1740	TSP45I
*M. monacense*	72 / 79 / 156 / 252 / 380 / 715 / 927 / 1046 / 1266	TSP45I
*M. marseillense*	855 / 1201 / 1342 / 1349	TSP45I
*M. mucogenicum*	164 / 303 / 344 / 482 / 554 / 770 / 798 / 1466	BsaWI
*M. noviomagense*	381 / 427 / 547 / 930 / 1108 / 1345	TSP45I
*M. novum*	123 / 163 / 374 / 540 / 755 / 797 / 914 / 1168	BsaWI
*M. nebraskense*	123/ 164/ 361/ 798/ 2263	BsaWI
*M. neoaurum*	156 / 188 / 250 / 928 / 1344 / 2009	TSP45I
*M. immunogenum*	94 / 156 / 194 / 250 / 928 / 979 / 1051 / 1111	TSP45I
*M. intracellulare*	308 / 452 / 854 / 889 / 980 / 1349	TSP45I
*M. ostraviense*	123 / 164 / 225 / 373 / 798 / 1421 / 1643	BsaWI
*M. rufum*	79 / 383 / 406 / 630 / 636 / 690 / 929 / 1115	TSP45I
*M. rutilum*	156 / 250 / 357 / 383 / 630 / 648 / 715 / 763 / 929	TSP45I
*M. paragordonae*	164 / 494 / 554 / 799 / 2424	BsaWI
*M. paraintracellulare*	308 / 452 / 854 / 889 / 893 / 1349	TSP45I
*M. paratuberculosis*	129/ 350/ 1140/ 1626/ 1748	TSP45I
*M. pallens*	368 / 381 / 640 / 929/ 1117 / 1343	TSP45I
*M. paraseoulense*	95 / 495 / 798 / 1350 / 1858	BsaWI
*M. paraterrae*	79 / 422 / 596 / 928 / 1268 / 1488	TSP45I
*M. parmense*	199 / 210 / 306 / 929 / 1340 / 1739	TSP45I
*M. senriense*	308 / 856 / 893 / 1341 / 1349	TSP45I
*M. saskatchewanense*	123 / 164 / 225 / 361 / 396 / 799 / 1005 / 1627	BsaWI
*M. senegalense*	121/ 164 / 195 / 460 / 554 / 799/ 958 / 1510	BsaWI
*M. septicum*	73 / 156 / 250 / 426 / 620 / 929 / 1038 / 1345	TSP45I
*M. shigaense*	199 / 209 / 371 / 929 / 1343 / 1650	TSP45I
*M. smegmatis*	73/ 156 / 254 / 383 / 652 / 929 / 1044 / 1347	TSP45I
*M. spongiae*	211 / 308 / 1140 / 1344 / 1749	TSP45I
*M. seoulense*	164 / 495 / 810 / 1420/ 1859	BsaWI
*M. shinjukuense*	110 / 198 / 1344 / 1349 / 1759	TSP45I
*M. shottsii*	452 / 700 / 1100 / 2042	TSP45I
*M. sinensis*	110 / 420 / 537 / 630 / 930 / 964 / 1471	TSP45I
*M. simiae*	199 / 209 / 929 / 1342 / 2034	TSP45I
*M. stephanolepidis*	96 / 113 / 156 / 194 / 250 / 326 / 639 / 815 / 1051 / 1111	TSP45I
*M. stomatepiae*	408 / 929 / 1340 / 2034	TSP45I
*M. tuberculosis*	96/ 110/ 198/ 211/ 1139/ 1248 / 1768	TSP45I
*M. thermoresistibile*	156 / 257/ 307/ 381/ 618 / 666/ 928/ 1347	TSP45I
*M. ulcerans*	452 / 892 / 1348 / 2043	TSP45I
*M. vaccae*	75 / 156 / 170 / 252 / 381 / 529 / 630 / 640 / 929 / 1114	TSP45I
*M. vanbaalenii*	156 / 172 / 252 / 382 / 515 / 929 / 1114 / 1345	TSP45I
*M. vicinigordonae*	121 / 164 / 373 / 471 / 554 / 799 / 2264	BsaWI

### In silico validation of the proposed PCR-RFLP assay

Initial validation of the specificity of selected primer sets for *Mycobacterium* species revealed the amplification of selected targets from other non-*Mycobacterium* genera. The primer set with the lowest number of cross-matches with nonmycobacterial genera was selected (Table S3). Out of examined 5,064 examined genomes, the selected primer set amplified the specific target from 119 genomes, including the 75 investigated mycobacterial genomes (Additional File S1). The phylogenetic analysis of amplified selected targets from these 119 genomes (Figure S1) revealed that 26 non-mycobacterial species from three distinct genera(*Mycolicibacterium*, *Mycolicibacter*, and *Mycobacteroides*) belong to the same family of *Mycobacterium* named *Mycobacteriaceae*^27^. In fact, they were previously classified as different species under the same genus of *Mycobacterium*, but recently, they were reclassified as different genera^28^. We did not include these 26 genomes in our database because they are not clinically relevant. However, we also validated the restriction pattern profiles for these 26 species and ensured that they had distinct patterns from those of our 75 *Mycobacterium* species (Figure S2, Figure S3). Additionally, we identified 18 genomes that were not related to *Mycobacteriaceae*. We validated that these non-*Mycobacteriaceae* genomes have different restriction profiles from our database of selected *Mycobacterium* species restriction profiles as well (Table S4).

Using additional 942 whole genomes of different reference *Mycobacterium* spp. from the NCBI assembly database, the selected primer set was found in all 942 genomes that were recruited, and the virtually amplified sequences were able to make an in silico profile using TSP45I or BsaWI restriction enzymes (Table S6). Among the recruited genomes, 632 *M. tuberculosis* genomes showed 100% similarity with the in silico profiles. For *M. bovis and M. avium*, the recruited genomes included 42 and 78 whole genomes, respectively. Upon validation, all species were 100% similar to the proposed in silico profiles of *M. bovis* and *M. avium*. Finally, the remaining 190 genomes of other *Mycobacterium* species exhibited similar profiles to the proposed in silico profiles that matched their designated species upon recruitment.

Additional validation using raw sequencing files for mycobacterial isolates deposited in the ENA database revealed the ability of the proposed assay to generate the same pattern for each species as in the pattern database (Table S7). This validation step reinforced the reliability and accuracy of the primers for subsequent PCR amplification and analysis. This comprehensive analysis of a large dataset of patient assembly genome files thoroughly evaluated the integrity of the proposed in silico PCR-RFLP assay.

## Discussion

In response to the significant limitations of previously described molecular tools for the differential identification of clinically relevant mycobacterial species, the present study describes a novel in silico tool that use PCR-RFLP technique. The proposed assay provides precise and comprehensive differentiation power to discriminate between various mycobacterial species, including previously unresolved closely related species. Previously described tools included MicroSeq 500 16S rDNA and MycoAlign methods that can discriminate between *Mycobacterium* species based on the sequences of 16S RNA and ITS regions, respectively^11,13^. However, these methods are time-consuming and have a potential limited accuracy because they depend on the sequencing and sequence analysis of amplified targets, which requires specialized laboratory equipment and expertise. The INNO-LiPA MYCOBACTERIA v2 system is another assay that can discriminate between at least 18 different mycobacterial species^12^. This system uses a different technique known as the line probe assay to identify DNA variations among various bacterial species. The limited number of available probes and the use of a high-cost membrane containing multiple probes for each tested sample have made the assay unpractical for routine diagnostic work in clinical laboratories because of its high expenses and limited coverage. On the other hand, our proposed assay, besides having more discriminating power between larger numbers of *Mycobacterium* species, is also less costly and time-saving compared to previously mentioned assays as it relies on specific target amplification followed by digestion with specific restriction enzymes to generate species-specific RFLP patterns.

Similar to our PCR-RFLP approach for the proposed assay, PRA-hsp65 and rpoB Duplex PCR and hsp65 PCR-RFLP are other molecular diagnostic assays that use similar approaches^14,15^. However, compared with our assay, which utilizes a single PCR target and only two restriction enzymes for the generation of highly discriminating species-specific RFLP patterns, these assays utilize multiple PCR targets with more than one restriction enzyme for each target, which makes them more time-consuming and costly compared with our proposed assay.

All previously described molecular assays for the differential identification of *Mycobacterium* species can discriminate between MTBC and NTM and provide for the differential identification of several *Mycobacterium* species. However, none of these assays could discriminate between members of the MTBC group. We attributed this to the high homology of previously used targets (16S, ITS1, and ITS2, hsp65) among members of MTBC^8^ compared with the more hypervariable target used in our proposed assay. In this context, it is worth mentioning that differentiation between *M. bovis* and *M. tuberculosis*, two members of the MTBC group, is crucial for tuberculosis treatment regimens^8^. *M. bovis*, a cause of human tuberculosis, is naturally resistant to pyrazinamide, a first-line drug for tuberculosis that reduces the treatment period from up to 18 months to only 6 months^29^. Other tests that can be used currently include traditional culture and biochemical analysis^30^, which takes a lot of time. Alternstively, more expensive molecular methods can be used, like sequencing and sequence analysis of specific targets for mutation detection of the pncA gene, which is the target gene of pyrazinamide Interestingly. Yet the method is time consuming, more expensive and not very accurate as it could be affected by machine sequencing errors. The currently proposed assays provide a unique method for the clear differential identification of MTBC members, such as *M. tuberculosis*,* M. bovis*,* M. africanum*,* M.* *microti*, *M. caprae*, and *M. canettii*, which other competing assays have failed to resolve (Fig. 4).

The main limitation of the proposed assay is the lengthy target (~ 5 kb). PCR amplification of such large targets requires a special protocol for amplification^31^. Additionally, enzymatic restriction analysis will also require a special protocol to avoid the star activity effect, where the enzyme can digest the DNA sequence at an unrecognized sequence^32^. Therefore, the proposed assay requires further laboratory study to optimize suitable protocols before clinical validation.

## Conclusion

This in silico approach based on the RFLP is a promising method for accurately identifying mycobacterial species. This approach overcomes the limitations associated with traditional diagnostic methods and molecular techniques by leveraging genomic analysis to identify specific primers and restriction enzymes. The primers and restriction enzymes identified in this study have potential for the reliable identification of various mycobacterial species. The proposed system can discriminate between the most clinically important *Mycobacterium* species, including those of MTBC. However, the proposed system is in silico and requires further protocol optimization for laboratory validation. The proposed assay can be used in epidemiological studies to improve clinical diagnostics and guide treatment decisions. Further validation and implementation of this approach are necessary to establish its effectiveness in clinical and public health settings. By enabling rapid and accurate identification, this in silico approach can significantly contribute to the management and control of mycobacterial infections.

## Electronic supplementary material

Below is the link to the electronic supplementary material.

Supplementary Material 1

Supplementary Material 2

Supplementary Material 3

## Data Availability

All data generated or analyzed during this study are included in this published article and its supplementary information files. The Python code used to find candidate primer sets with restriction enzymes is available on GitHub https://github.com/mahmoudkotb5/MycoSeq-RPA.

## Abbreviations

ENA: European Nucleotide Archive
GTF: Gene transfer format
ITS: Internal transcribed spacer
MTBC: *Mycobacterium tuberculosis* complex
NCBI: National Center for Biotechnology Information
NTM: Non-tuberculous mycobacteria
PCR: Polymerase chain reaction
RFLP: Restriction fragment length polymorphism
TB: Tuberculosis
Tm: Melting temperature
WGS: Whole-genome sequencing
