# Supplementary material for: A novel in silico molecular tool for comprehensive differentiation of Mycobacterium species
Source: Sci Rep. 2025 Feb 10;15:4981. doi: 10.1038/s41598-025-89148-0 (PMC11810988; doi:10.1038/s41598-025-89148-0)
Supplement: Supplementary file 1 — Supplementary Material 1 [file 41598_2025_89148_MOESM1_ESM.pdf]

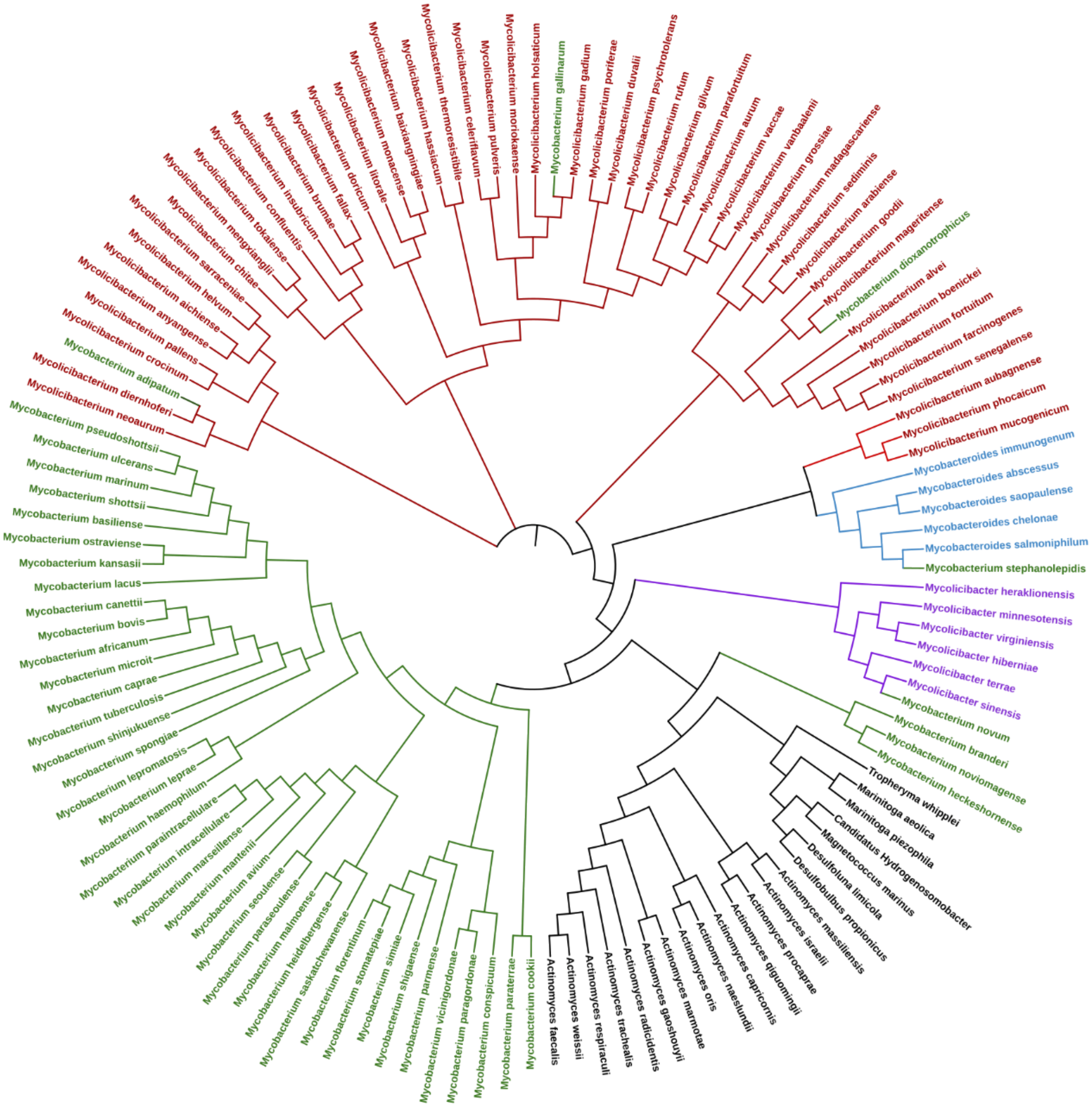

Figure S1. Phylogenetic tree for all 119 targets amplified using the selected primer set.

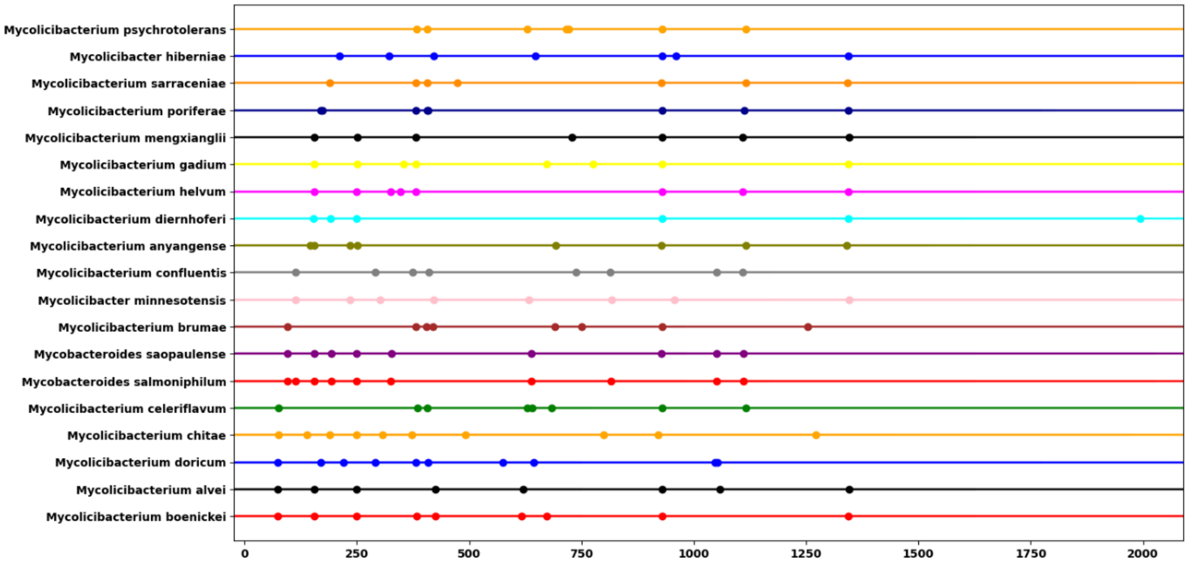

Figure S2. The TSP45I restriction profile for 19 *Mycolicibacterium, Mycolicibacter* and *Mycobacteroides* genomes that are not in our restriction database.

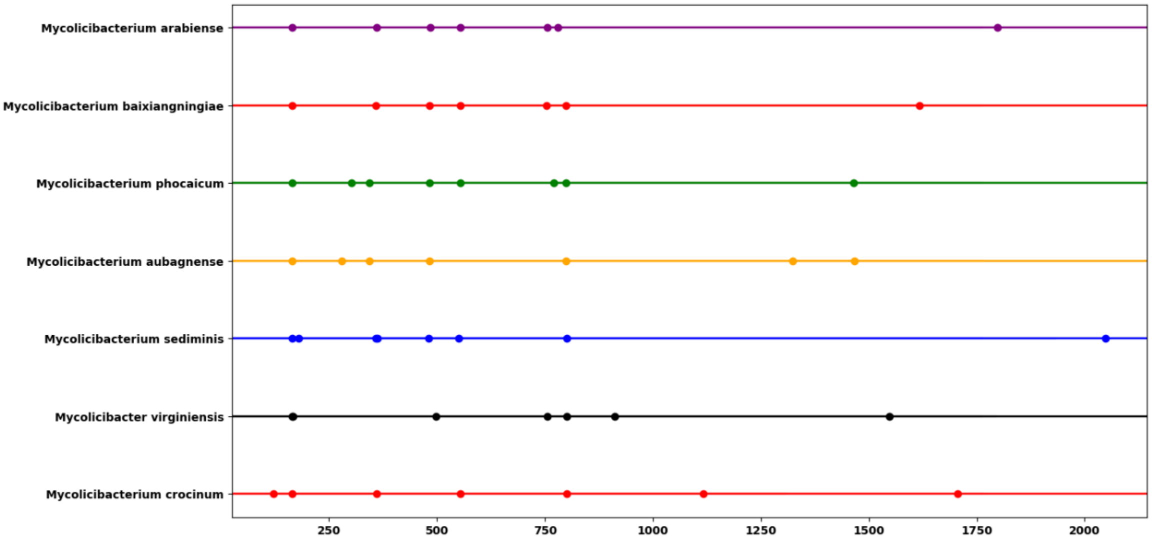

Figure S3. The BsaWI restriction profile for 7 *Mycolicibacterium, Mycolicibacter and Mycobacteroides* genomes that are not in our restriction database.
